# Zipf's Law in Short-Time Timbral Codings of Speech, Music, and Environmental Sound Signals

**DOI:** 10.1371/journal.pone.0033993

**Published:** 2012-03-29

**Authors:** Martín Haro, Joan Serrà, Perfecto Herrera, Álvaro Corral

**Affiliations:** 1 Music Technology Group, Universitat Pompeu Fabra, Barcelona, Spain; 2 Artificial Intelligence Research Institute (IIIA-CSIC), Consejo Superior de Investigaciones Científicas, Bellaterra, Barcelona, Spain; 3 Complex Systems Group, Centre de Recerca Matemàtica, Bellaterra, Barcelona, Spain; University of Zaragoza, Spain

## Abstract

Timbre is a key perceptual feature that allows discrimination between different sounds. Timbral sensations are highly dependent on the temporal evolution of the power spectrum of an audio signal. In order to quantitatively characterize such sensations, the shape of the power spectrum has to be encoded in a way that preserves certain physical and perceptual properties. Therefore, it is common practice to encode short-time power spectra using psychoacoustical frequency scales. In this paper, we study and characterize the statistical properties of such encodings, here called timbral code-words. In particular, we report on rank-frequency distributions of timbral code-words extracted from 740 hours of audio coming from disparate sources such as speech, music, and environmental sounds. Analogously to text corpora, we find a heavy-tailed Zipfian distribution with exponent close to one. Importantly, this distribution is found independently of different encoding decisions and regardless of the audio source. Further analysis on the intrinsic characteristics of most and least frequent code-words reveals that the most frequent code-words tend to have a more homogeneous structure. We also find that speech and music databases have specific, distinctive code-words while, in the case of the environmental sounds, this database-specific code-words are not present. Finally, we find that a Yule-Simon process with memory provides a reasonable quantitative approximation for our data, suggesting the existence of a common simple generative mechanism for all considered sound sources.

## Introduction

Heavy-tailed distributions (e.g. power-law or log-normal) pervade data coming from processes studied in several scientific disciplines such as physics, engineering, computer science, geoscience, biology, economics, linguistics, and social sciences [1]–[6]. This ubiquitous presence has increasingly attracted research interest over the last decades, specially in trying to find a unifying principle that links and governs such disparate complex systems [5]–[17]. Even though this unifying principle has not been found yet, major improvements in data analysis and engineering applications have already taken place thanks to the observation and characterization of such heavy-tailed distributions. For instance, research on statistical analysis of natural languages [18] facilitated applications such as text retrieval based on keywords, where the word probability distributions are used to determine the relevance of a text to a given query [19]. A particularly important landmark was the seminal work of Zipf [6], showing a power-law distribution of word-frequency counts with an exponent 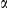 close to 1,
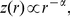
(1)where 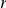 corresponds to the rank number (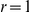 is assigned to the most frequent word) and 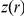 corresponds to the frequency value of the word with rank 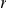. The rank-frequency power-law described by Zipf (Eq. 1) also indicates a power-law probability distribution of word frequencies [3],
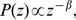
(2)where 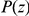 is the probability mass function of 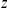 and 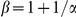.

Zipf himself reported power-law distributions in other domains, including melodic intervals and distances between note repetitions from selected music scores [6]. Since then, several works have shown heavy-tailed distributions of data extracted from symbolic representations of music such as scores [20], [21] and MIDI files [22]–[24] (MIDI is an industry standard protocol to encode musical information; this protocol does not store sound but information about musical notes, durations, volume level, instrument name, etc.). However, unlike text retrieval, sound retrieval has not directly benefited from such observations yet [25]. Indeed, symbolic representations are only available for a small portion of the world's music and, furthermore, are non-standard and difficult to define for other types of sounds such as human speech, animal vocalizations, and environmental sounds. Hence, it is relevant to work directly with information extracted from the raw audio content. In this line, some works can be found describing heavy-tailed distributions of sound amplitudes from music, speech, and crackling noise [2], [26], [27].

Sound amplitudes refer to air pressure fluctuations which, when being digitized, are first converted into voltage and then sampled, quantized, and stored in digital format as discrete time series. Sound amplitude correlates with the subjective sensation of *loudness*, which is one of the three primary sensations associated with sound perception [28]. The other two pillars of sound perception are *pitch*, which correlates with the periodicity of air pressure fluctuations, and *timbre*, which mainly correlates with the audio waveform shape and, thus, with the spectro-temporal envelope of the signal (i.e. the temporal evolution of the shape of the power spectrum) [28]. According to the American National Standards Institute “timbre is that attribute of auditory sensation in terms of which a listener can judge that two sounds similarly presented and having the same loudness and pitch are dissimilar” [29]. Thus, timbre is a key perceptual feature that allows to discriminate between different sounds. In particular, it has been shown that “timbre is closely related to the relative level produced at the output of each auditory filter [or critical band of hearing]” [30] (in the auditory filter model, the frequency resolution of the auditory system is approximated by a bank of band-pass filters with overlapping pass-bands). Moreover, it is common practice in audio technological applications to quantitatively characterize timbral sensations by encoding the energy of perceptually motivated frequency bands found in consecutive short-time audio fragments [31], [32].

In the present work we study and characterize the statistical properties of encoded short-time spectral envelopes as found in disparate sound sources. In the remainder of the paper we will pragmatically refer to such encoded short-time spectral envelopes as timbral code-words. We are motivated by the possibility that modeling the rank-frequency distribution of timbral code-words could lead to a much deeper understanding of sound generation processes. Furthermore, incorporating knowledge about the distribution of such code-words would be highly beneficial in applications such as similarity-based audio retrieval, automatic audio classification, or automatic audio segmentation [31]–[33].

Here, we study 740 hours of four different types of real-world sounds: *Speech*, *Western Music*, *non-Western Music*, and *Sounds of the Elements* (the latter referring to sounds of natural phenomena such as rain, wind, and fire; see **Materials & Methods**). We observe and characterize the same heavy-tailed (Zipfian) distribution of timbral code-words in all of them. This means that the different short-time spectral envelopes are far from being equally probable and, instead, there are a few that occur very frequently and many that happen rarely. Furthermore, given Eq. 1, there is no characteristic separation between these two groups. We find that this heavy-tailed distribution of timbral code-words is not only independent of the type of sounds analyzed; it seems also independent of the encoding method, since similar results are obtained using different settings. Our results also indicate that regardless of the analyzed database, the most frequent timbral code-words have a more homogeneous structure. This implies that for frequent code-words, proximate frequency bands tend to have similar encoded values. We also describe timbral code-word patterns among databases. In particular, the presence of database-specific timbral code-words in both speech and music, and the absence of such distinctive code-words for *Sounds of the Elements*. Finally, we find that the generative model proposed by Cattuto et al. (which is a modification of the Yule-Simon model) [13] provides a reasonable quantitative account for the observed distribution of timbral code-words, suggesting the existence of a common generative framework for all considered sound sources.

### General Procedure

As mentioned, short-time spectral envelopes are highly related to the perception of timbre, one of the fundamental sound properties. In order to characterize the distribution of these spectral envelopes, we first need an appropriate way of numerically describing them. Next, we need to quantize each spectro-temporal description in such a manner that similar envelopes are assigned to the same encoded type. This allows us to count the number of tokens corresponding to each type (i.e. the frequency of use of each envelope type). Ultimately, each of these types can be seen as a code-word assigned from a predefined dictionary of timbres. We now give a general explanation of this process (more details are provided in **Materials & Methods**).

**Figure 1:**
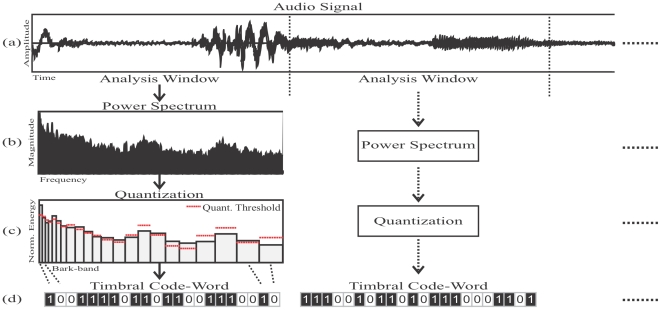
Block diagram of the encoding process. a) The audio signal is segmented into non-overlapping analysis windows. b) The power spectrum of the audio segment is computed. c) The shape of the power spectrum is approximated by Bark-bands. d) Each Bark-band is binary-quantized by comparing the normalized energy of the band against a pre-computed energy threshold. These 22 quantized bands from a timbral code-word.

We represent the timbral characteristics of short-time consecutive audio fragments following standard procedures in computational modeling of speech and music [31]–[33]. First, we cut the audio signal into non-overlapping temporal segments or analysis windows (Fig. 1a). Then, we compute the power spectrum of such audio segment (Fig. 1b). Next, we approximate the overall shape (or envelope) of the power spectrum by computing the relative energy found in perceptually motivated bands (Fig. 1c). Finally, we quantize each band by comparing its energy against a stored energy threshold (red lines in Fig. 1c). In particular, if the band's value is smaller than the band's threshold we encode this band as “0”, otherwise we encode it as “1” (Fig. 1d).

We consider three perceptually motivated window sizes, namely: 46, 186, and 1,000 ms. The first one (46 ms) is selected because it is extensively used in audio processing algorithms and tries to capture the small-scale nuances of timbral variations [32], [33]. The second one (186 ms) corresponds to a perceptual measure for sound grouping called “temporal window integration” [34], usually described as spanning between 170 and 200 ms. Finally, we explore the effects of a relatively long temporal window (1 s) that exceeds the usual duration of speech phonemes and musical notes. For the perceptually motivated bands of the power spectrum we use a well-known auditory scale of frequency representation that emulates the frequency response of the human cochlea, namely, the Bark scale [35]. From this process we obtain one timbral representation per temporal window, corresponding to the so-called energy-normalized Bark-bands [36]. This timbral representation is formed by a real-valued vector of 22 dimensions per window, reflecting the percentage of energy contained in each frequency band between 0 and 9,500 Hz (i.e. the first 22 critical bands of hearing). Such an upper bound is motivated by the fact that most of the perceptually relevant sounds lie below this threshold [28] and because adding more bands exponentially multiplies the computational load of our experiments.

For the quantization process we first estimate, from a representative sample of sounds, the median value per each component of the 22-dimensional vector (i.e. the value that splits each dimension into two equally populated regions). These median values are stored as quantization thresholds and used to binary-quantize each Bark-band vector. This binary quantization roughly resembles the all-or-none behavior of neurons and neuronal ensembles [37]. As mentioned, we encode each temporal window as a sequence of 22 zeros and ones. Thus, the total amount of possible code-words (i.e. the encoding dictionary) is 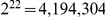 timbral code-words. This encoding method is akin to methods used, for instance, in automatic audio identification [38] or in cochlear implant sound processors [39].

**Figure 2:**
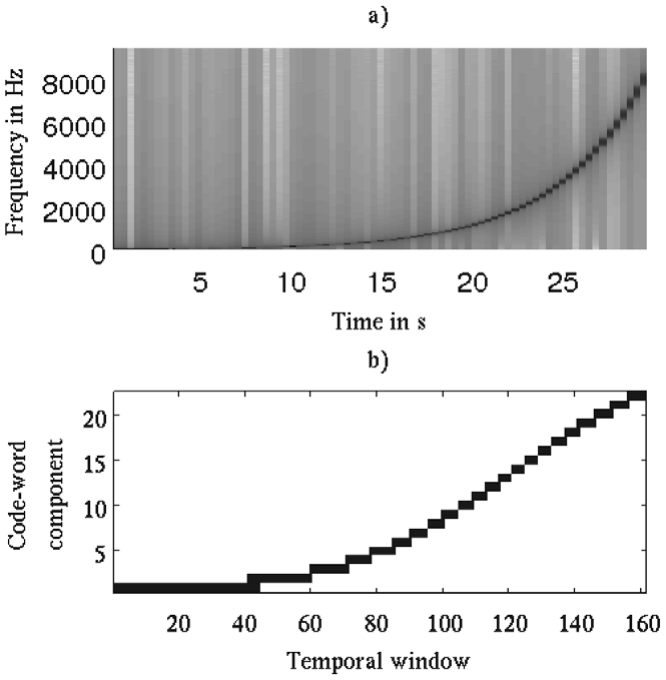
Spectrogram vs. timbral code-word example. a) Spectrogram representation for a sinusoidal sweep in logarithmic progression over time going from 0 to 9,500 Hz. The color intensity represents the energy of the signal (white = no energy, black = maximum energy). This standard representation is obtained by means of the short-time Fourier transform. b) Timbral code-word representation of the same audio signal. The horizontal axis corresponds to temporal windows of 186 ms and the vertical axis shows the quantized values per Bark-band (black = 1 and white = 0). For instance, in the first 40 temporal windows only the first Bark-band is quantized as one (the first Bark-band corresponds to frequencies between 0 and 100 Hz). A total of 37 different code-words are used to encode this sinusoidal sweep.

As an illustrative example, Fig. 2a shows the time-frequency representation of a sinusoidal sweep in logarithmic progression over time, ranging from 0 to 9,500 Hz. Fig. 2b shows the resulting timbral code-words for the same piece of audio. In both plots we can see the sweeping of the sinusoidal sound. Thus, we can observe how the timbral code-words form a simplified representation of the spectral content of the signal while preserving the main characteristics of its spectral shape (the difference between both curve shapes is due to the use of different frequency representations; the spectrogram uses a linear frequency representation while timbral code-words are computed using a non-linear scale based on psychoacoustical findings). As a further example, we consider the number of distinct timbral code-words used to encode sounds with disparate timbral characteristics, ranging from a simple sinusoidal wave up to multi-instrument polyphonic music (Table 1). As expected, we observe a positive correlation between the timbral “richness” of the analyzed sounds and the number of code-words needed to describe them (i.e. as the timbral variability increases, sounds are encoded using a greater number of different code-words).

**Table 1:** Number of different timbral code-words used to describe each sound.

Sound Description	# code-words
Sine wave 440 Hz	1
Rain	18
1/f (Pink) Noise	26
White Noise	28
Sinusoidal Sweep (0–9,500 Hz)	37
Clarinet solo	97
Female English speaker	128
String Quartet	135
Voice, Drums, Bass & Synth. Strings	140
Philharmonic Orchestra	141
Voice and Electronic Instruments	153

Examples computed from 30 s audio files using an analysis window of 186 ms (160 temporal windows in total). Pink and white noise sounds were generated using Audacity (http://audacity.sourceforge.net). **String Quartet** corresponds to a rendition of F. Haydn's Op.64 No. 5 “The Lark”, **Voice, Drums, Bass & Synth. Strings** corresponds to Michael Jackson's *Billie Jean*, **Philharmonic Orchestra** corresponds to a rendition of *The Blue Danube* by J. Strauss II, and **Voice and Electronic Instruments** corresponds to Depeche Mode's *The world in my eyes*.

## Results

### Zipfian Distribution of Timbral Code-Words

**Figure 3:**
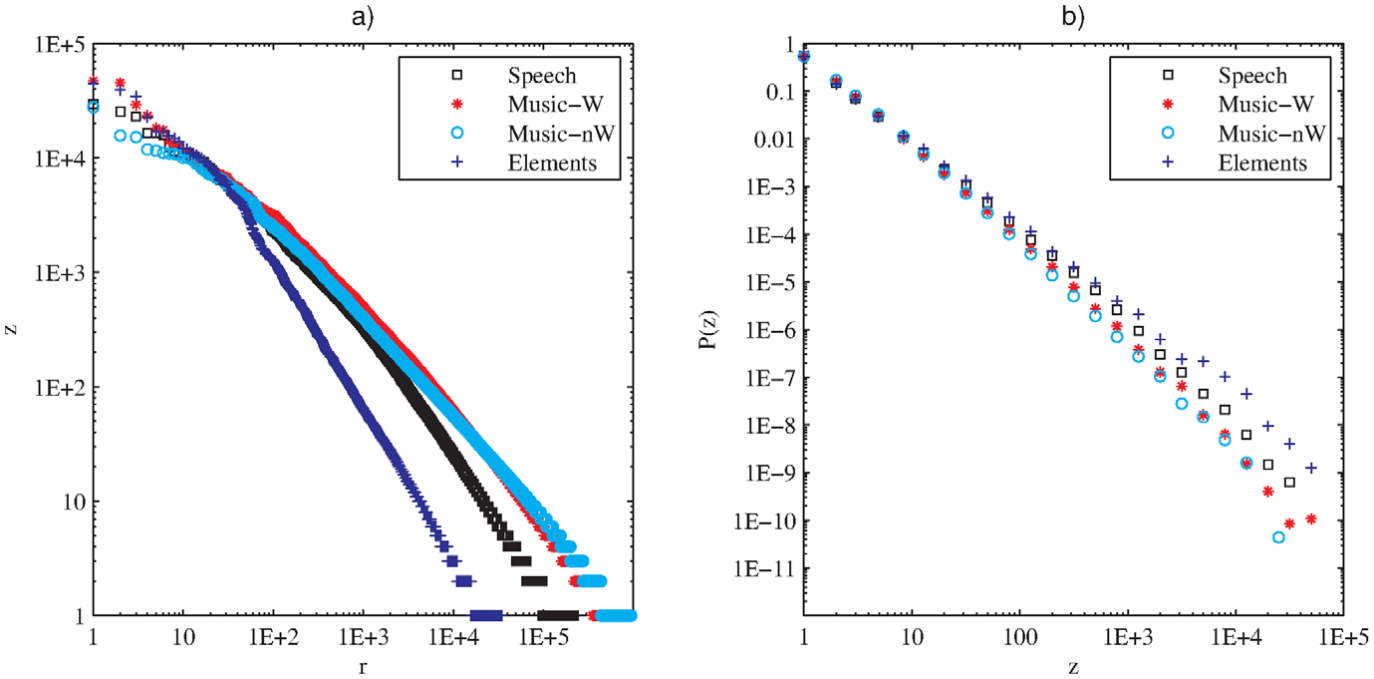
Timbral code-words encoded from Bark-bands. a) Rank-frequency distribution of timbral code-words per database (encoded Bark-bands, analysis window = 186 ms). b) Probability distribution of frequencies for the same timbral code-words. Music-W means *Western Music*, Music-nW means *non-Western Music* and Elements means *Sounds of the Elements*.

For each database we count the frequency of use of each timbral code-word (i.e. the number of times each code-word is used) and sort them in decreasing order of frequency (Fig. 3a). We find that a few timbral code-words are very frequent while most of them are very unusual. In order to evaluate if the found distribution corresponds to a Zipfian distribution, instead of working directly with the rank-frequency plots we focus on the equivalent description in terms of the distribution of the frequency (Fig. 3b). Maximum-likelihood estimation of the exponent, together with the Kolmogorov-Smirnov test are used for this purpose [40], [41] (see **Materials & Methods**). In all cases we obtain that a power-law distribution is a good fit beyond a minimum frequency 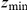. Moreover, consistently with Zipf's findings in text corpora, all the estimated Zipfian exponents are close to one (Table 2). The high frequency counts for few timbral code-words are particularly surprising given the fact that we used a very large coding dictionary (recall that each temporal window was assigned to one out of more than four million possible code-words).

**Table 2:** Power-law fitting results for Bark-band code-words per database and window size.

DB/Window	N words	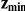	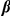	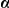
Speech				
46 ms	494,926	2,000	2.20 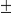 .05	0.84 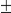 .04
186 ms	219,595	501	2.22 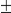 .05	0.82 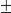 .03
1,000 ms	100,273	79	2.33 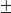 .05	0.75 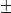 .03
Music-W				
46 ms	1,724,245	2,000	2.26 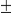 .04	0.79 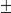 .03
186 ms	798,871	794	2.33 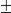 .06	0.75 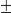 .03
1,000 ms	240,236	79	2.29 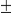 .03	0.78 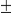 .02
Music-nW				
46 ms	1,905,444	126	2.17 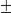 .01	0.85 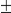 .01
186 ms	947,327	50	2.17 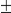 .01	0.85 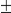 .01
1,000 ms	306,682	5	2.17 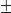 .01	0.86 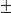 .01
Elements				
46 ms	125,248	794	1.95 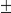 .04	1.05 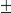 .05
186 ms	34,171	20	1.79 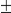 .02	1.27 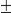 .03
1,000 ms	10,231	8	1.79 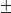 .02	1.27 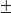 .03

**DB/Window** means database name and window size, **N words** is the number of used code-words, 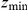 is the minimum frequency for which the Zipf's law is valid, 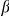 is the frequency-distribution exponent (Eq. 2), and 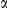 corresponds to the Zipf's exponent (Eq. 1).

Regarding text corpora, it has been recently shown that simple random texts do not produce a Zipfian distribution [42]. In the case of our timbral code-words it would be non-trivial to generate random sequences that resemble a Zipf's law-like rank distribution. All our code-words have the same length (22 characters) and are formed by two possible characters (“0” and “1”). Since our quantization thresholds correspond the median values found in a representative database, the probability of occurrence of each character in our experiments is close to 0.5. Therefore, if we generate a random sequence of words formed by 22 binary characters having similar probability of occurrence we would observe similar word counts for all generated random words. Thus, the shape of the rank-frequency distribution for those random words would be close to a horizontal line (i.e. slope close to zero). Only in extreme cases where the probability of occurrence of one character is much higher than the other we will observe long tailed rank-frequency distributions, but, even in those cases, the distribution will differ from a real Zipfian distribution. Instead of being a straight line in the log-log plot it would present a staircase shape. In the utmost case of one character having probability one, only one word (a sequence of 22 equal characters) will be repeatedly generated producing a delta-shaped rank distribution (note that in our encoding scenario, a delta-shaped rank distribution would be produced if the analyzed database contains only one static sound, like in the case of the sine wave encoded in Table 1).

**Figure 4:**
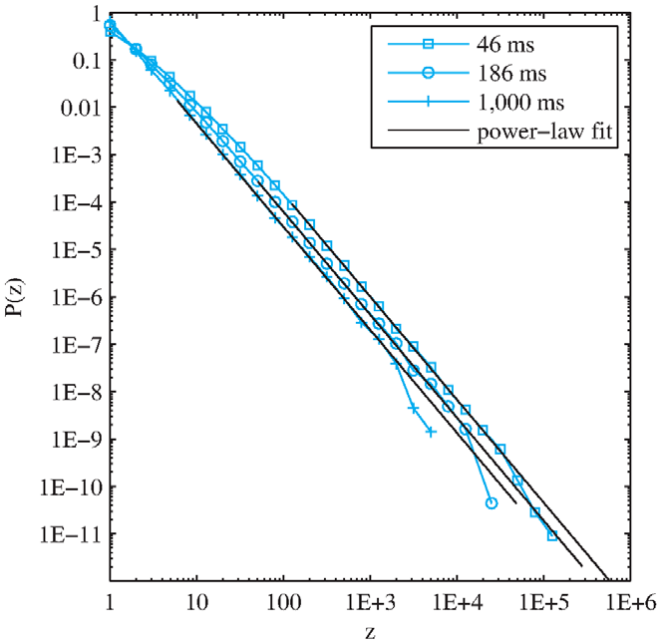
Probability distribution of frequencies of timbral code-words for *non-Western Music* analyzed with window sizes of 46, 186, and 1,000 ms.

We now study the robustness of the found distribution against the length of the analysis window. Remarkably, changing the analysis window by almost one and a half orders of magnitude (from 46 to 1,000 ms) has no practical effect on the estimated exponents. This is especially valid for *Speech* and both *Western* and *non-Western Music* databases. Fig. 4 shows an example of the probability distribution of frequencies and the estimated power-laws for timbral code-words of *non-Western Music* analyzed with the three considered temporal windows (46, 186, and 1,000 ms). The main effect produced by changing the window size seems to be that the smaller the window, the larger the minimum frequency value from which the power-law is found to be a plausible fit for the data (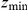 in Table 2).

We further investigate the robustness of the rank-frequency distributions by re-computing the code-words while altering some parts of the encoding process. Since we are describing the spectro-temporal envelopes using a psychoacoustical scale (the Bark scale) and, given that psychoacoustical scales present higher resolution (i.e. small bandwidth) in the low frequency ranges, we re-compute the code-words using 22 equally-spaced frequency bands (431.8 Hz each). The obtained results are very similar to those obtained using Bark-bands (see **Supporting Information S1**). This suggests that similar results would be obtained for other psychoacoustical scales like the Mel scale [43] or the ERB scale [44]. We also tested several quantization thresholds, extracted from a sample of different database combinations, without observing any significant change in the rank-frequency plots. Finally, since our encoding process includes a pre-processing step that in order to emulate the sensitivity of the human ear, filters the signal according to an equal-loudness curve (see **Materials & Methods**), we re-computed the whole process without this equal-loudness filter. In this case the obtained results were practically identical to the ones obtained using the equal-loudness filter.

Another interesting fact with regard to the distribution's robustness is that when analyzing the rank-frequency counts of timbral code-words of randomly selected audio segments of up to 6 minutes in length (a duration that includes most of the songs in Western popular music), a similar heavy-tailed distribution as the one found for the whole databases is observed (see **Supporting Information S1**). This behavior, where similar distributions are found for medium (i.e. a few minutes) and long-time (i.e. many hours) code-word sequences, further supports the robustness of the found distribution.

The evidence presented in this section suggests that the found Zipfian distribution of timbral code-words is not the result of a particular type of sound source, sound encoding process, analysis window, or sound length, but an intrinsic property of the short-time spectral envelopes of sound.

### Timbral Code-Word Analysis

**Figure 5:**
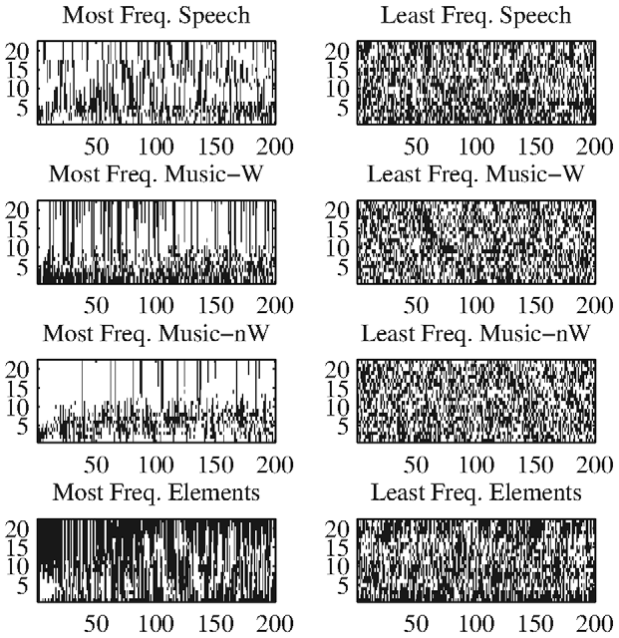
Most (left) and least (right) frequent timbral code-words per database (window size = 186 ms). The horizontal axis corresponds to individual code-words (200 most common and a random selection of 200 of the less common). The vertical axis corresponds to quantized values per Bark-band (white = 0, black = 1). Every position in the abscissa represents a particular code-word.

**Figure 6:**
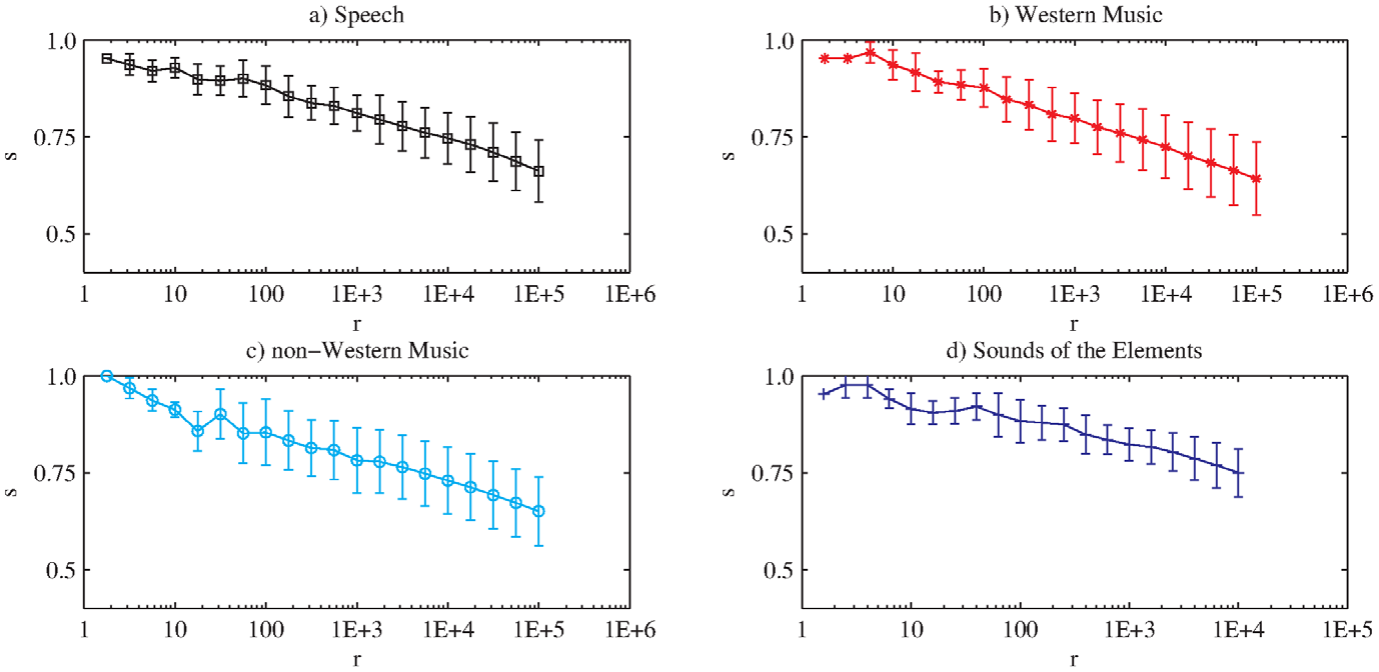
Smoothness values (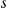) per database. For a better visualization we plot the mean and standard deviation of the smoothness value of 20 logarithmically-spaced points per database (window size = 186 ms).

We now provide further insight into the specific characteristics of timbral code-words, as ordered by decreasing frequency. In particular, when we examine their inner structure, we find that in all analyzed databases the most frequent code-words present a smoother structure, with close Bark-bands having similar quantization values. Conversely, less frequent elements present a higher band-wise variability (Fig. 5). In order to quantify this smoothness, we compute the sum of the absolute values of the differences among consecutive bands of a given code-word (see **Materials & Methods**). The results show that all databases follow the same behavior, namely, that the most frequent timbral code-words are the smoother ones. Thus, the smoothness value tends to decrease with the rank (see Fig. 6).

Next, we analyze the co-occurrence of timbral code-words between databases (see also **Supporting Information S1**). We find that about 80% of the code-words present in the *Sounds of the Elements* database are also present in both *Western* and *non-Western Music* databases. Moreover, 50% of the code-words present in *Sounds of the Elements* are also present in *Speech*. There is also a big overlap of code-words that belong to *Western* and *non-Western Music* simultaneously (about 40%). Regarding the code-words that appear in one database only, we find that about 60% of the code-words from *non-Western Music* belong exclusively to this category. The percentage of database-specific code-words in *Western Music* lies between 30 and 40% (depending on the window size). In the case of the *Speech* database, this percentage lies between 10 and 30%. Remarkably, the *Sounds of the Elements* database has almost no specific code-words.

We also find that within each database, the most frequent timbral code-words were temporally spread throughout the database. Therefore, their high frequency values are not due to few localized repetitions. In fact, we observe local repetitions of frequent code-words across the whole database (see **Supporting Information S1**). Finally, we find that the largest number of different timbral code-words used by the four databases was 2,516,227 (window size = 46 ms). Therefore there were 1,678,077 timbral code-words (40% of the dictionary) that were never used (i.e. more than 1.5 million Bark-band combinations that were not present in 740 hours of sound).

### Generative Model

When looking for a plausible model that generates the empirically observed distribution of timbral code-words we have taken into consideration the following characteristics of our data. First, our timbral code-words cannot be seen as communication units like in the case of musical notes, phonemes, or words (although a sequence of short-time spectral envelopes constitutes one of the relevant information sources used in the formation of auditory units [45]). Second, we have here found the same distribution for processes that involve a sender and a receiver (like in speech and music sounds) and for processes that do not involve an intelligent sender (like inanimate environmental sounds). Therefore, we do not consider generative models that imply a communication paradigm, or any kind of intentionality or information interchange between sender and receiver (e.g. like in the case of the “least effort” model [6], [11]).

**Figure 7:**
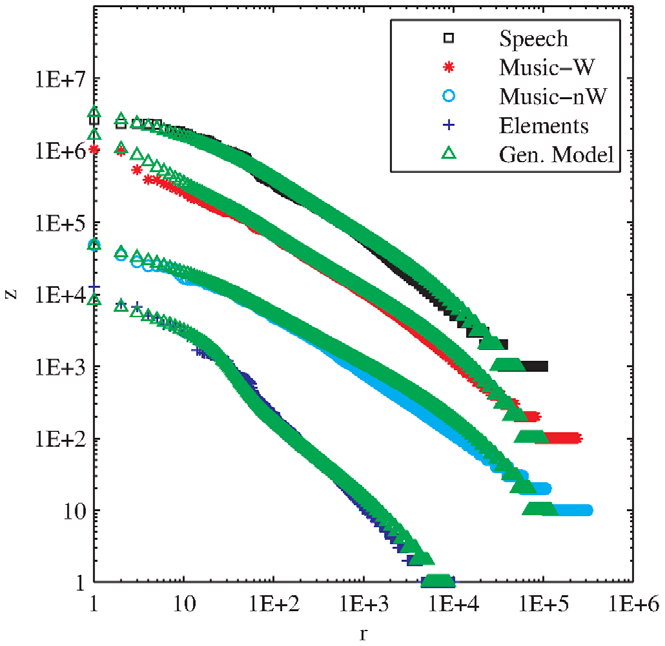
Rank-frequency distribution of timbral code-words (window = 1,000 ms) and Yule-Simon model with memory [**13**] per database. *Gen. Model* stands for the computed generative model. For clarity's sake the curves for *non-Western Music*, *Western Music*, and *Speech* are shifted up by one, two, and three decades respectively. The model's parameters 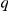, 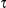, and 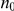 were manually adjusted to match the experimental data. They correspond to the probability of adding a new code-word, the memory parameter, and the number of initial code-words respectively. The adjusted parameters are 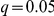, 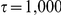, and 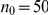 for *Sounds of the Elements*; 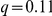, 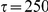, and 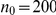 for *Speech*; 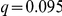, 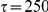, 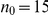 for *Western Music* and 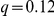, 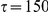, and 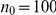 for *non-Western Music*. All model's curves were computed by averaging 50 realizations with identical parameters.

As for the generative models that do not imply intentionality, we have first considered the simple Yule-Simon model [7]. In this model, at each time step, a new code-word is generated with constant probability 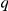, whereas an existing code-word is uniformly selected with probability 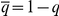. However, in preliminary analysis, this generative model did not provide a good fit to our data. Next, we explored the histogram of inter code-word distances for the 20 most frequent code-words per database (the inter code-word distance is just the number of code-words found between two identical and consecutive code-words plus one; see **Supporting Information S1**). From these plots we can see that, in general, the most frequent inter code-word distances correspond to short time gaps. This behavior leads us to consider the model proposed by Cattuto et al. [13]. This model modifies the original Yule-Simon model by introducing a hyperbolic memory kernel that when selecting an existing word, it promotes recently added ones thus favoring small time gaps between identical code-words. That is, instead of choosing uniformly from past words, this model selects a past word that occurred 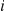 time steps behind with a probability that decays with 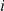 as 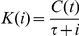, where 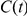 is a normalization factor and 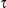 is a characteristic time-scale over which recent words have similar probabilities. When considering this modified Yule-Simon model a reasonable fitting is observed for the rank-frequency distributions (Fig. 7).

## Discussion

In the present article we have analyzed the rank-frequency distribution of encoded short-time spectral envelopes coming from disparate sound sources. We have found that these timbral code-words follow a heavy-tailed distribution characterized by Zipf's law, regardless of the analyzed sound source. In the light of the results presented here, this Zipfian distribution is also independent of the encoding process and the analysis window size. Such evidence points towards an intrinsic property of short-time spectral envelopes, where a few spectral shapes are extremely repeated while most are very rare.

We have also found that the most frequent code-words present a smoother structure, with neighboring spectral bands having similar quantization values. This fact was observed for all considered sound sources. Since most frequent code-words have also small inter code-word distances, it seems clear that these frequent code-words can be described as presenting both band-wise correlations and temporal recurrences. All this suggests that, as in the case of text corpora [11], the most frequent code-words are also the least informative ones. Informative in the sense of information theory's self-information concept, where the self-information (or surprisal) 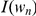 of a code-word 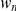 is defined as 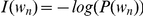, where 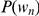 is the probability of occurrence of the code-word. Therefore, the bigger the code-word's probability, the smaller its self-information.

Our study also shows the presence of database-specific code-words for all databases except for *Sounds of the Elements*. This suggests that these natural sounds have been incorporated, possibly by imitation, within the human-made “palette” of timbres. Noticeably, it has been recognized that human vocal imitation, which is central to the human language capacity, has received insufficient research attention [46]. Moreover, a recent work [47] has suggested a mechanism by which vocal imitation naturally embeds single sounds into more complex speech structures. Thus, onomatopoeic sounds are transformed into the speech elements that minimize their spectral difference within the constraints of the vocal system. In this context, our observations could be taken as supporting the role of imitation within language and music evolution.

The fact that 40% of our dictionary remained unused after 740 hours of sounds suggests that this dictionary was big enough to accommodate the different timbral variations present in the databases, but it also poses the question about the reasons for this behavior. It could be that the unused spectral envelopes were unlikely (in physical-acoustical terms) or, perhaps, that animal sounds and urban soundscapes (the two large categories that have not been included in our study) would account for that.

We have also found that the modified version of the Yule-Simon generative model proposed by Cattuto et al. [13] provides a good quantitative approximation of our data. This model implies a fundamental role of temporally close events and suggests, in our case, that when repeating pre-occurred timbres, those that have occurred recently have more chance to reappear. This simple generative mechanism could possibly act as universal framework for the generation of timbral features. In particular, we know that the analyzed sounds are formed by mixtures of individual sources (e.g. notes simultaneously played by several musical instruments). Most of these individual sources can be modeled by an excitation-resonance process [28]. That is, an excitative burst (or series of bursts) of decaying energy that goes through biological or physical structures that impose certain acoustic properties on the original spectrum of the burst (e.g. the spectrum of the burst produced by the vocal folds is modulated/filtered by the shape of the vocal tract). Thus, the intrinsic characteristics of this resonance structure will favor the close reappearance of certain types of spectral envelopes every time the resonance structure is excited. This temporally close reappearance is properly reproduced by the modified Yule-Simon model.

In the light of our findings, the establishment of Zipf's law seems to be a physical property of the spectral envelopes of sound signals. Nevertheless, the existence of such scale-invariant distribution should have some influence on the way perception works because the perceptual-motor system reflects and preserves the scale invariances found in the statistical structure of the world [48]. Following this line of thought, we hypothesize that any auditory system, being natural or artificial, should exploit the here-described distribution and characteristics of short-time spectral envelopes in order to achieve an optimal trade-off between the amount of extracted timbral information and the complexity of the extraction process. Furthermore, the presented evidence could provide an answer to the question posed by Bregman in his seminal book *Auditory Scene Analysis* [45]:

[…] the auditory system might find some utility in segregating disconnected regions of the spectrum if it were true in some probabilistic way that the spectra that the human cares about tend to be smoothly continuous rather than bunched into isolated spectral bands.

According to our findings, these smoothly continuous spectra correspond to the highly frequent elements in the power-law distribution. We expect this highly repeated elements to quickly provide general information about the perceived sources (e.g. is it speech or music?). On the other hand, we expect that the rare spectral envelopes will give information about specific characteristics of the sources (e.g. the specific type of guitar that is being perceived).

Since we have found similar distributions for medium-time (i.e. a few minutes) than for long-time (i.e. many hours) code-word sequences, this behavior has direct practical implications that we would like to stress. One practical implication is that when selecting random short-time audio excerpts (using a uniform distribution), the big majority of the selected excerpts will belong to the most frequent code-words. Therefore, the knowledge extracted from such data sample will represent these highly frequent spectral envelopes but not necessary the rest of the elements. For instance, this is the case in two recently published papers [49], [50] where the perception of randomly selected short-time audio excerpts was studied. Moreover, auditory gist perception research [51] could also benefit from knowing that spectral envelopes are heavy-tailed distributed.

Another area on which the found heavy-tailed distributions will have practical implications is within audio-based technological applications that work with short-time spectral envelope information. For instance, in automatic audio classification tasks it is common practice to use an aggregated spectral envelope as timbral descriptor. That is, all the short-time spectral envelopes that form an audio file are aggregated into one mean spectral envelope. This mean envelope is then used to represent the full audio file, e.g. one song. This procedure is usually called the bag-of-frames method by analogy with the bag-of-words method used in text classification [52]. Evidently, computing statistical aggregates, like mean, variance, etc. on a set that contains highly frequent elements will be highly biased towards the values of this elements. In audio similarity tasks, the similarity between two sounds is usually estimated by computing a distance measure between sequences of short-time spectral envelope descriptors [53], e.g. by simply using the Euclidean distance. Again, these computations will be highly biased towards those highly frequent elements. Therefore, the influence this biases have on each task should be thoroughly studied in future research. It could be the case that for some applications considering only the most frequent spectral envelopes is the best solution. But, if we look at other research areas that deal with heavy-tailed data we can see that the information extracted from the distribution's tail is at least, as relevant as the one extracted from the most frequent elements [18], [54].

Finally, the relationship between the global Zipfian distribution present in long-time sequences, and the local heavy-tailed distributions depicted by medium-time sequences should be also studied. For instance, in text information retrieval, these type of research has provided improved ways of extracting relevant information [19]. Therefore, it is logical to hypothesize that this will be also the case for audio-based technological applications.

## Materials and Methods

### Databases

The *Speech* database is formed by 130 hours of recordings of English speakers from the *Timit* database (Garofolo, J S et al., 1993, “TIMIT Acoustic-Phonetic Continuous Speech Corpus”, Linguistic Data Consortium, Philadelphia; about 5.4 hours), the *Library of Congress* podcasts (“Music and the brain” podcasts: http://www.loc.gov/podcasts/musicandthebrain/index.html; about 5.1 hours), and 119.5 hours from *Nature* podcasts (http://www.nature.com/nature/podcast/archive.html; from 2005 to April 7th 2011, the first and last 2 minutes of sound were removed to skip potential musical contents). The *Western Music* database is formed by about 282 hours of music (3,481 full tracks) extracted from commercial CDs accounting for more than 20 musical genres including: rock, pop, jazz, blues, electronic, classical, hip-hop, and soul. The *non-Western Music* database contains 280 hours (3,249 full tracks) of traditional music from Africa, Asia, and Australia extracted from commercial CDs. Finally, in order to create a set that clearly contrasted the other selected ones, we decided to collect sounds that were not created to convey any message. For that reason we gathered 48 hours of natural sounds produced by natural inanimate processes such as water sounds (rain, streams, waves, melting snow, waterfalls), fire, thunders, wind, and earth sounds (rocks, avalanches, eruptions). This *Sounds of the Elements* database was gathered from the *The Freesound Project* (http://www.freesound.org). The differences in size among databases try to account for their differences in timbral variations (e.g. the sounds of the elements are less varied, timbrically speaking, than speech and musical sounds; therefore we can properly represent them with a smaller database.)

### Encoding Process

In order to obtain the timbral code-words we follow the same encoding process for every sound file in every database. Starting from the time-domain audio signal (digitally sampled and quantized at 44,100 Hz and 16 bits) we apply an equal-loudness filter. This filter takes into account the sensitivity of the human ear as a function of frequency. Thus, the signal is filtered by an inverted approximation of the equal-loudness curves described by Fletcher and Munson [55]. The filter is implemented as a cascade of a 10th order Yule-Walk filter with a 2nd order Butterworth high-pass filter [56].

Next, the signal is converted from the time domain to the frequency domain by taking the Fourier transform on non-overlapped segments [56] (using a Blackman-Harris temporal window) of either 46, 186, or 1,000 ms length (2,048, 8,192, and 44,100 audio samples, respectively). From the output of the Fourier transform we compute its power spectrum by taking the square of the magnitude. The Bark-band descriptor is obtained by adding up the power spectrum values found between two frequency edges defined by the Bark scale. Since we want to characterize timbral information regardless of the total energy of the signal, we normalize each Bark-band value by the sum of all energy bands within each temporal window. The output of this process is a sequence of 22-dimensional vectors that represents the evolution of the signal's spectral envelope. The used Bark-band frequency edges are: 0, 100, 200, 300, 400, 510, 630, 770, 920, 1,080, 1,270, 1,480, 1,720, 2,000, 2,320, 2,700, 3,150, 3,700, 4,400, 5,300, 6,400, 7,700, and 9,500 Hz [35].

After having computed the energy-normalized Bark-band descriptors on a representative database we store the median value of each dimension and window size. This way, each dimension is split into two equally populated groups (median splitting). The representative database contains all Bark-band values from the *Sounds of the Elements* database plus a random sample of Bark-band values from the *Speech* database that matches in number the ones from the *Sounds of the Elements*. It also includes random selections of *Western Music* and *non-Western Music* matching half of the length of *Sounds of the Elements* each. Thus, our representative database has its Bark-bands values distributed as one third coming from *Sounds of the Elements*, one third from *Speech*, and one third from *Music* totaling about 20% of the whole analyzed sounds. We constructed 10 of such databases per analysis window and, for each dimension, we stored the mean of the median values as representative median (see **Supporting Information S1**). Finally, we quantize each Bark-band dimension by assigning all values below the stored threshold to “0” and those being equal or higher than the threshold to “1”. After this quantization process every temporal window is mapped into one of the 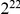 possible timbral code-words.

### Power-Law Estimation

To evaluate if a power-law distribution holds we take the frequency of each code-word as a random variable and apply up-to-date methods of fitting and testing goodness-of-fit to this variable [40], [41]. The procedure consists in finding the frequency range 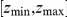 for which the best power-law fit is obtained. First, arbitrary values for lower and upper cutoffs 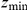 and 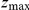 are selected and the power-law exponent 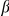 is obtained by maximum-likelihood estimation. Second, the Kolmogorov-Smirnov test quantifies the separation between the resulting fit and the data. Third, the goodness of the fit is evaluated by comparing this separation with the one obtained from synthetic simulated data (with the same range and exponent 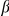) to which the same procedure of maximum-likelihood estimation plus Kolmogorov-Smirnov test is applied, which yields a 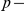
value as a final result. Then, the procedure selects the values of 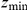 and 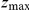 which yield the largest log-range 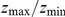 provided that the 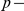
value is above a certain threshold (for instance 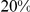). See **Supporting Information S1** for details. In all cases we have obtained that we can take 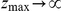 and results with finite 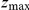 are not presented here.

### Code-Word Smoothness

The code-word smoothness 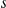 was computed using
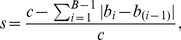
(3)where 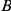 corresponds to the number of bands per timbral code-word (22 in our case), 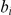 corresponds to the value of band 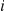 and 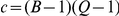, where 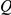 corresponds to the number of quantization steps (e.g. 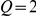 for binary quantization).

## Supporting Information

Supporting Information S1 **Supporting information regarding: quantization thresholds, code-words extracted from equally-spaced frequency bands, temporal distribution of timbral code-words, rank-frequency distribution of medium-length audio excerpts, timbral code-word co-occurrence, inter code-word distance, and power-law fitting procedure.**
(PDF)
