# Supplementary material for: Zipf's Law in Short-Time Timbral Codings of Speech, Music, and Environmental Sound Signals
Source: PLoS One. 2012 Mar 29;7(3):e33993. doi: 10.1371/journal.pone.0033993 (PMC3315504; doi:10.1371/journal.pone.0033993)
Supplement: Supporting Information S1 — Supporting information regarding: quantization thresholds, code-words extracted from equally-spaced frequency bands, temporal distribution of timbral code-words, rank-frequency distribution of medium-length audio excerpts, timbral code-word co-occurrence, inter code-word distance, and power-law fitting procedure. (PDF) [file pone.0033993.s001.pdf]

Supporting Information: Zipf's law in short-time timbral codings of speech, music, and environmental sound signals

Martín Haro[1,*], Joan Serrà[1,2], Perfecto Herrera[1], Álvaro Corral[3]
1 Music Technology Group, Universitat Pompeu Fabra, Roc Boronat 138, 08018 Barcelona, Spain.
2 Artificial Intelligence Research Institute (IIIA-CSIC), Consejo Superior de Investigaciones Científicas, Campus de la UAB s/n, 08193 Bellaterra, Barcelona, Spain.
3 Complex Systems Group, Centre de Recerca Matemàtica, Edifici Cc, Campus Bellaterra, 08193 Bellaterra, Barcelona, Spain.
∗ E-mail: martin.haro@upf.edu

Quantization Thresholds

Table 1 shows the median values per Bark-band and window size. These median values are used to binary-quantize the energy-normalized Bark-bands (see main text).

Code-words from Equally-Spaced Frequency Bands

Fig. 1 shows the rank-frequency distribution and the probability distribution of frequencies of timbral code-words encoded with equally-spaced frequency bands. Table 2 shows the fitting results for the equally-spaced encodings from the different databases and window sizes. From these results we can see that the obtained Zipfian distributions are similar to those obtained with code-words constructed using the Bark scale (see main text). Noticeably, this time, all Zipf's exponents are bigger than one and they are stable for the two small temporal windows only (except for *non-Western Music* were all window sizes share almost the same exponent).

Temporal Distribution of Timbral Code-Words

In Fig. 2 an example of the temporal distribution of the most frequent timbral code-words that account for 20 % of the *non-Western Music* database is shown. As it can be seen, the code-words are temporally spread throughout the entire time axis. This implies that the high frequency counts are not due to few localized repetitions. The same temporal spreading was observed for the most frequent timbral code-words found in the rest of the databases and for different window sizes.

Rank-Frequency Distribution of Medium-Length Audio Excerpts

In Fig. 3, an example of rank-frequency distributions of randomly selected audio excerpts per database can be seen. In the case of *Western* and *non-Western Music* databases the excerpts correspond to individual songs. In the case of *Speech* and *Sounds of the Elements* the audio files were cut with arbitrary lengths of up to 6 min. From the plots we can observe that these medium-length sounds also present a heavy-tailed distribution similar to that observed with the full databases.

Timbral Code-Word Co-occurrence

Tables 3, 4, 5 and 6 show the number of co-occurring timbral code-words as obtained with the 186 ms window. These tables account for co-occurrence of code-words that describe 20, 50, 80 and 100 % of each database, respectively.

Inter Code-Word Distance

Fig. 4 shows the inter code-word distance for the 100 most frequent timbral code-words per database.

Power-Law Fit

It is important to provide evidence that our frequency distributions are well fit by power laws. The reason to work with frequency distributions is that the frequency can be considered as a random variable, whereas the rank is not. In order to perform the fits we use maximum likelihood (ML) estimation [1]. For a continuous random variable Z, following a power-law distribution given by the probability density,

$$f(z) = \frac{\beta - 1}{1 - j^{\beta-1}} \frac{a^{\beta-1}}{z^\beta} \propto \frac{1}{z^\beta},$$

defined in the range $a \leq Z \leq b$ and with $j = a/b$ (note that a and b correspond to z_{min} and z_{max} in the main text), the ML estimator of the exponent β is given by the maximization of the log-likelihood as a function of β,

$$\frac{\ln L}{N_{ab}} = \ln(\beta - 1) - \ln(1 - j^{\beta-1}) - (\beta - 1) \ln \frac{G_{ab}}{a} - \ln G_{ab},$$

where N_{ab} is the number of code-word types comprised between a and b and G_{ab} is the geometric mean of the frequencies on that range.

For a discrete random variable Z taking values $a, a+1, \ldots b-1, b$, it is easy to show, following Clauset et al. [1], that the log-likelihood in the power-law case is well approximated, in the limit of large a, by

$$\frac{\ln L}{N_{ab}} = \ln(\beta - 1) - \ln(1 - k^{\beta-1}) - (\beta - 1) \ln \frac{G_{ab}}{a - 1/2} - \ln G_{ab},$$

where $k = (a - 1/2)/(b + 1/2)$. This means that a discrete power-law distribution between a and b can be replaced by a continuous one between $a - 1/2$ and $b + 1/2$ if a is large enough, which in practice is usually achieved by $a \geq 5$ [1] if b is much larger than a. For a power-law with no upper limit ($b \to \infty$), the previous formula is still valid just taking $k = 0$ and therefore a closed formula can be obtained for β, which is given by $\beta = 1 + 1/[\ln G_{ab} - \ln(a - 1/2)]$.

For the error ε of the exponent β, we approximate the formula for the continuous case,

$$\sqrt{N_{ab}}\, \varepsilon = \left[\frac{1}{(\beta - 1)^2} - \frac{k^{\beta-1} \ln^2 k}{(1 - k^{\beta-1})^2} \right]^{-1/2},$$

which corresponds to one standard deviation of the distribution of β when N_{ab} is large. For $b \to \infty$, the limit $k = 0$ yields $\varepsilon = (\beta - 1)/\sqrt{N_{ab}}$.

A maximization of the likelihood does not guarantee a good fit if the probabilistic (power-law) model is not appropriate. It is necessary then to test the goodness of the fit. In the same way as Clauset et al. (and this choice is a matter of taste) we use the Kolmogorov-Smirnov (KS) test [2]. This is defined by the KS statistic, or KS distance, which is the maximum difference between the empirical cumulative distribution and the theoretical cumulative distribution corresponding to the ML fit, i.e.,

$$d_{KS} = \max_{\forall z_i} \left[S(z_i) - \frac{i}{N_{ab}} \right],$$

where i denotes the number of data equal or above z_i, z_i corresponds to a value taken by the variable Z, and $S(z_i)$, the survivor function of Z, is well approximated (for large a) by

$$S(z) = \frac{1}{1 - k^{\beta-1}} \left[\left(\frac{a - 1/2}{z - 1/2} \right)^{\beta-1} - k^{\beta-1} \right].$$

The value of the KS distance does not suffice to characterize the fit as good or bad; we need a scale in order to compare it. This scale is obtained by computer simulations of the resulting fitted ML power-law distribution, approximated, for reasonably large a, by

$$z = \left\lfloor \frac{a - 1/2}{[1 - u(1 - k^{\beta-1})]^{1/(\beta-1)}} + \frac{1}{2} \right\rfloor,$$

where u is a continuous uniform random number between 0 and 1 and $\lfloor ... \rfloor$ denotes the integer part of its argument (which therefore, with the term $+1/2$ inside, rounds the other term to the nearest integer). For a large number of synthetic data sets, with N_{ab} elements each, the same procedure as for the empirical data is repeated: ML estimation of the β exponent plus the calculation of the KS distance between each synthetic distribution and its fit. In this way, a distribution of KS distances is obtained under the null hypothesis that the data come from a power-law distribution. The $p-$value is then defined as the probability that for true power-law distributed data, as the synthetic sets we have generated, the KS distance is above the empirical value; this is computed as the number of synthetic data sets for which their KS distance is larger than the empirical one divided by the total number of synthetic data sets.

In principle, for fixed values of a and b, we obtain the ML value of the exponent β and an associated $p-$value. In practice, however, a and b are not known, and one needs a criterion to select the optimum ones. At this point we depart from the recipe provided by Clauset et al. [1, 3]. We repeat the previous procedure for many different values of a and b and select the ones which maximize the log-range of the data, b/a, provided that the corresponding $p-$value is high enough. We usually use a threshold value equal to 20 %. It is important to realize that the $p-$value of the whole procedure is not the one corresponding to the selected values of a and b. Computer simulations tell us that the former is a factor 2 or 3 smaller than the latter. Nevertheless, the precise calculation of the $p-$value is not relevant for our purposes.

It turns out that for the data analyzed in this paper the resulting values of b are always larger than the maximum value taken by the variable (i.e. no data are outside the power-law range from the right side) and therefore it is simpler to assume $k = 0$ in the previous formulas and just work with a non-upper truncated power-law.

References

1. Clauset A, Shalizi CR, Newman MEJ (2009) Power-law distributions in empirical data. SIAM Review 51: 661.

2. Press WH, Teukolsky SA, Vetterling WT, Flannery BP (1992) Numerical recipes in FORTRAN. Cambridge University Press, Cambridge, 2 edition.

3. Corral A, Font F, Camacho J (2011) Non-characteristic half-lives in radioactive decay. Phys Rev E 83: 066103.

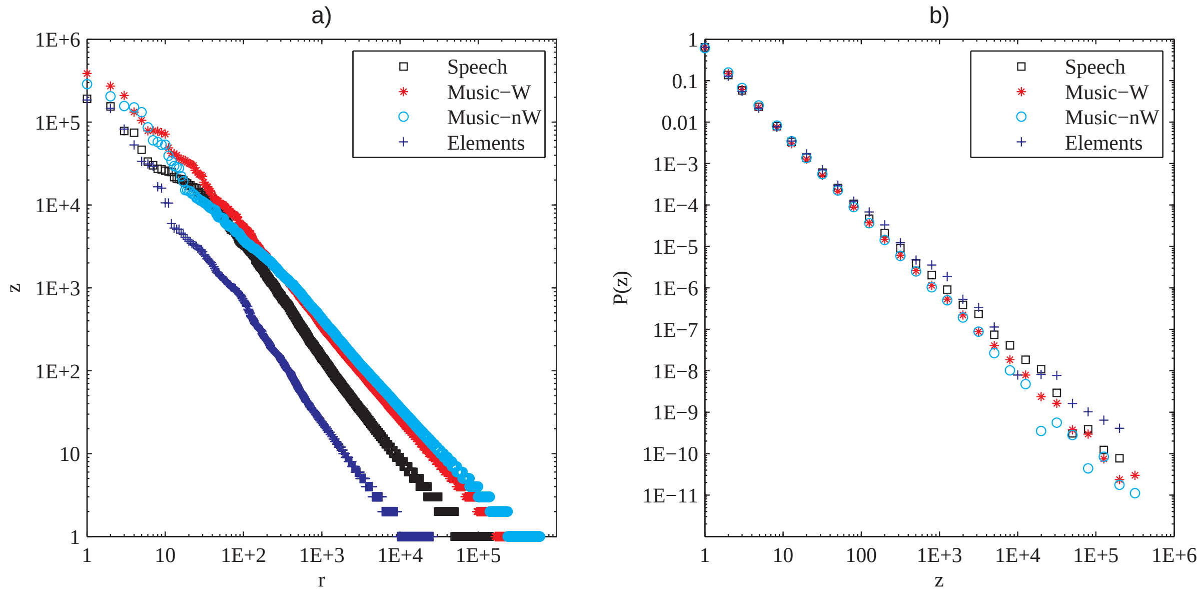

Figure 1. Timbral code-words encoded from equally-spaced frequency bands. a) Rank-frequency distribution of timbral code-words encoded from equally-spaced frequency bands (Bandwidth = 431.84 Hz, analysis window = 186 ms). b) Probability distribution of frequencies for the same timbral code-words.

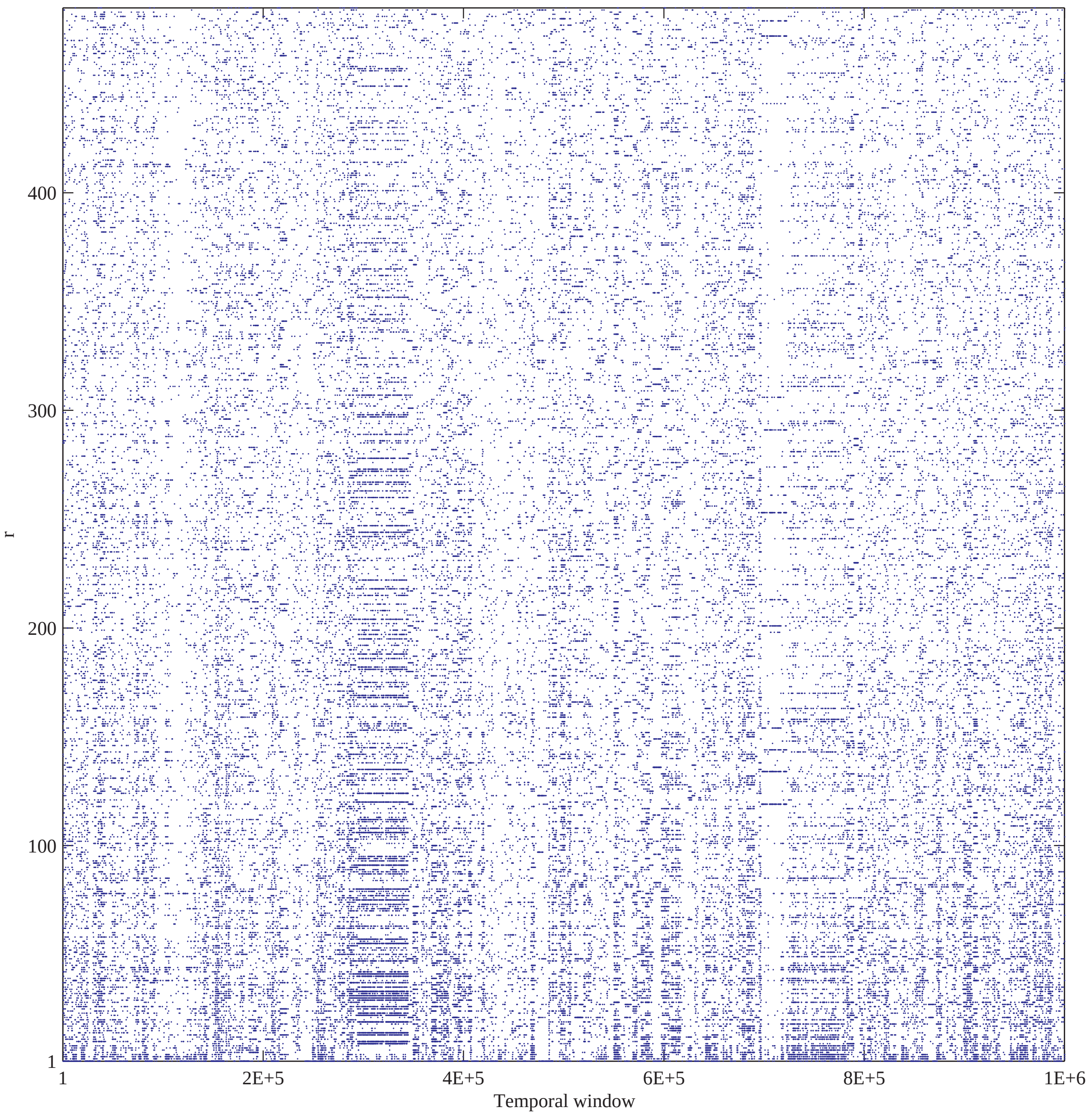

Figure 2. Temporal distribution of 485 most frequent code-wods in *non-Western Music* (window = 1,000 ms). Each dot indicates the temporal location (x axis) of a particular timbral code-word (y axis).

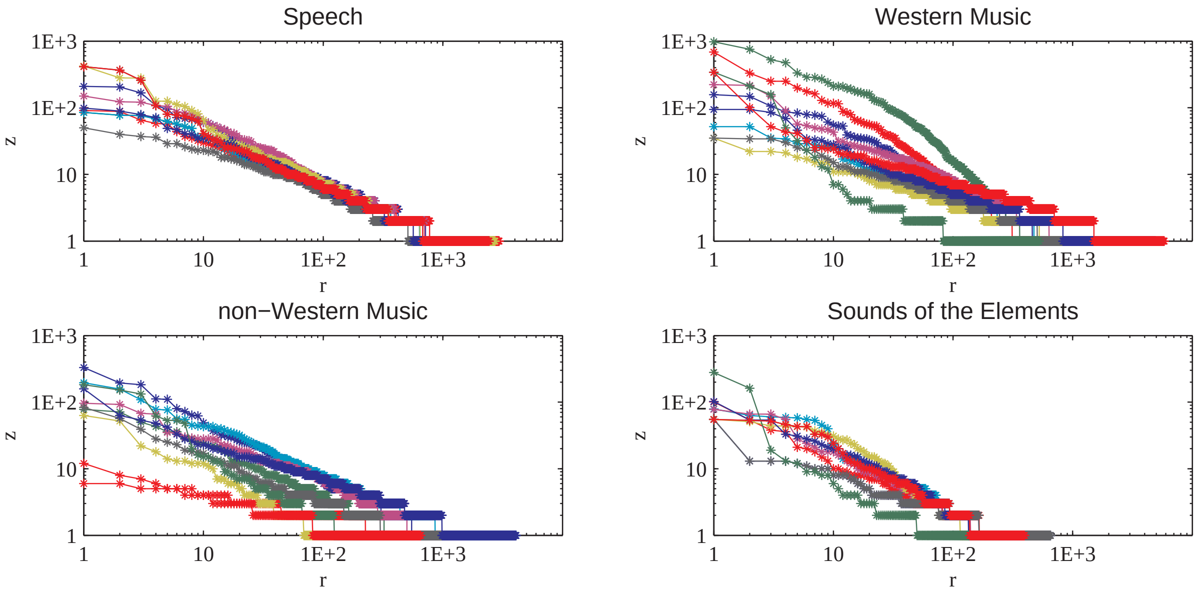

Figure 3. Rank-frequency distributions of timbral code-words from ten randomly selected audio excerpts per database (Bark-bands, window = 46 ms). In the case of *Western Music* and *non-Western Music* each line corresponds to one song. In the case of *Speech* and *Sounds of the Elements* each line corresponds to an arbitrary audio segment of up to 6 min in length.

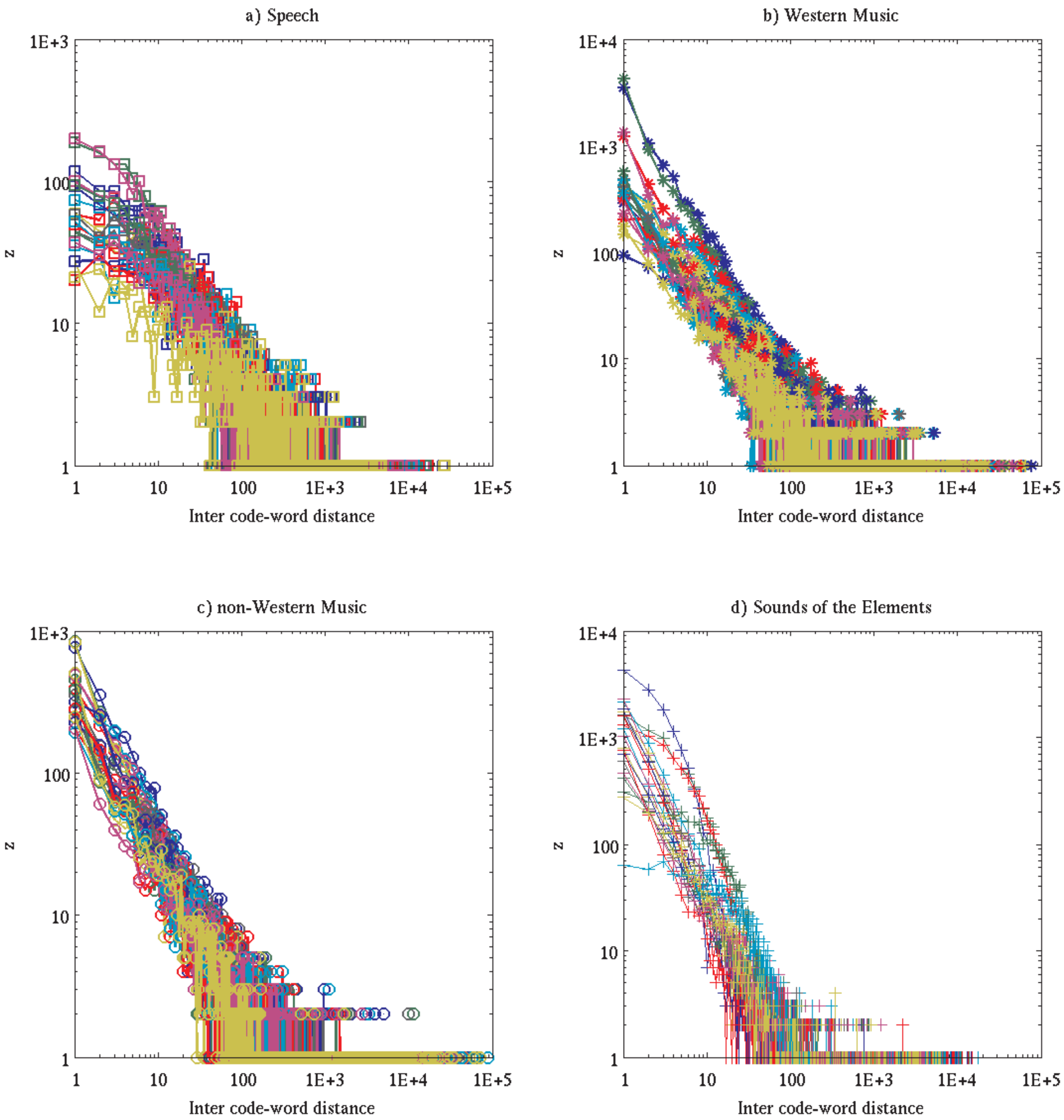

Figure 4. Inter code-word distance for the 20 most frequent code-word per database (Bark-bands, window = 1,000 ms).

Table 1. Median values used to quantize the energy-normalized Bark-bands.

Bark-band	Window size		
number	46 ms	186 ms	1,000 ms
1	6.19E-04	4.67E-04	4.45E-04
2	1.62E-02	2.37E-02	3.11E-02
3	5.04E-02	5.13E-02	5.95E-02
4	4.45E-02	4.29E-02	5.66E-02
5	3.08E-02	3.93E-02	5.17E-02
6	1.91E-02	2.52E-02	3.86E-02
7	1.69E-02	1.66E-02	2.48E-02
8	1.11E-02	1.19E-02	1.54E-02
9	7.79E-03	9.34E-03	1.08E-02
10	8.18E-03	8.90E-03	1.07E-02
11	8.02E-03	9.07E-03	1.09E-02
12	8.99E-03	1.09E-02	1.30E-02
13	1.13E-02	1.30E-02	1.47E-02
14	1.47E-02	1.56E-02	1.69E-02
15	2.15E-02	2.46E-02	2.61E-02
16	2.48E-02	2.56E-02	2.67E-02
17	2.83E-02	2.87E-02	2.99E-02
18	1.48E-02	1.61E-02	1.87E-02
19	5.35E-03	5.76E-03	7.65E-03
20	1.31E-03	1.42E-03	2.18E-03
21	2.63E-04	2.93E-04	4.79E-04
22	8.56E-05	9.86E-05	1.60E-04

Table 2. Power-law fitting results for code-words encoded from equally-spaced frequency bands per database and window size.

**DB/Window**	**N words**	$z_{\min}$	β	α
Speech				
46 ms	383,207	200	1.75± .02	1.33 ± .03
186 ms	139,452	79	1.74± .02	1.35 ± .03
1,000 ms	48,717	200	1.95± .06	1.05 ± .06
Music-W				
46 ms	1,288,416	126	1.91± .01	1.11 ± .01
186 ms	457,575	50	1.88± .01	1.14 ± .02
1,000 ms	103,364	32	1.80± .02	1.26 ± .03
Music-nW				
46 ms	1,514,576	50	1.97± .01	1.04 ± .01
186 ms	613,361	20	1.95± .01	1.05 ± .01
1,000 ms	175,518	79	1.98± .03	1.02 ± .03
Elements				
46 ms	111,593	50	1.77± .02	1.31 ± .03
186 ms	26,557	8	1.74± .02	1.35 ± .03
1,000 ms	5,453	20	1.70± .04	1.44 ± .08

DB/Window means database name and window size, **N words** is the number of used code-words, $z_{\min}$ is the minimum frequency for which the Zipf's law is valid, β is the frequency-distribution exponent, and α corresponds to the Zipf's exponent.

Table 3. Co-occurrence of code-words that account for 20 % of each database (window = 186 ms).

20 %			Music-nW	¬(Music-nW)
Music-W	Speech	Elements	0	0
		¬(Elements)	34	1
	¬(Speech)	Elements	5	0
		¬(Elements)	175	91
¬(Music-W)	Speech	Elements	0	0
		¬(Elements)	18	16
	¬(Speech)	Elements	1	0
		¬(Elements)	323	—

The symbol ¬() denotes the negation of the proposition inside the parentheses, e.g. ¬(Speech) stands for timbral code-words that do not belong to the Speech database.

Table 4. Co-occurrence of code-words that account for 50 % of each database (window = 186 ms).

50 %			Music-nW	¬(Music-nW)
Music-W	Speech	Elements	33	0
		¬(Elements)	523	6
	¬(Speech)	Elements	9	0
		¬(Elements)	4,434	2,253
¬(Music-W)	Speech	Elements	1	0
		¬(Elements)	328	154
	¬(Speech)	Elements	0	0
		¬(Elements)	11,784	—

Table 5. Co-occurrence of code-words that account for 80 % of each database (window = 186 ms).

80 %			Music-nW	¬(Music-nW)
Music-W	Speech	Elements	438	1
		¬(Elements)	8,906	280
	¬(Speech)	Elements	68	0
		¬(Elements)	60,471	41,477
¬(Music-W)	Speech	Elements	1	0
		¬(Elements)	2,992	847
	¬(Speech)	Elements	0	0
		¬(Elements)	121,154	—

Table 6. Co-occurrence of code-words that account for 100 % of each database (window = 186 ms).

100 %			Music-nW	¬(Music-nW)
Music-W	Speech	Elements	20,495	741
		¬(Elements)	93,674	21,912
	¬(Speech)	Elements	8,344	1,729
		¬(Elements)	291,497	360,478
¬(Music-W)	Speech	Elements	363	166
		¬(Elements)	38,101	44,142
	¬(Speech)	Elements	1,055	1,277
		¬(Elements)	493,797	—
